# Vitamin E‐stabilized polyethylene shows similar survival rates at minimum 7‐year follow‐up compared to conventional polyethylene in primary total knee arthroplasty

**DOI:** 10.1002/jeo2.12106

**Published:** 2024-09-05

**Authors:** Alessandro Bistolfi, Marco Spezia, Alessandra Cipolla, Monica Bonera, Danilo Mellano, Lorenzo Banci, Marta Colombo, Alessandro Massè

**Affiliations:** ^1^ Orthopaedics and Traumatology Cardinal Massaia Asti Hospital Asti Italy; ^2^ Centro Ortopedico Quadrante Hospital Omegna Italy; ^3^ University of the Studies of Turin Turin Italy; ^4^ Clinical Research Department Permedica Orthopaedics Merate Italy; ^5^ Department of Orthopaedics and Traumatology CTO AOU Città della Salute e della Scienza di Torino Turin Italy

**Keywords:** conventional polyethylene, total knee arthroplasty, UHMWPE, vitamin E, vitamin E‐stabilized

## Abstract

**Purpose:**

The aim of this cross‐sectional study was to compare survival, clinical and radiographic results of total knee arthroplasty (TKA) with vitamin E‐stabilized polyethylene (VEPE) or conventional polyethylene (CPE) at a minimum of 7‐year follow‐up.

**Methods:**

Patients who underwent primary TKA between 2011 and 2015, receiving the same cemented rotating platform knee design with VEPE or CPE tibial inserts, were identified. Patients were contacted for clinical and radiographic follow‐up. American Knee Society Score (KSS), Forgotten Joint Score (FJS‐12), presence of periprosthetic radiolucent lines (RLLs) and osteolysis were evaluated at the last follow‐up. Any revision, reintervention or other complications were recorded.

**Results:**

Among 350 TKAs initially identified, 102 VEPE and 97 CPE knees were included for analysis with mean follow‐up of 8.5 and 8.3 years, respectively. No significant difference was found in survival rates at 10‐year follow‐up with revision due to aseptic loosening (95.0% vs. 97.8%, *p* = 0.29) or due to any reason (87.6% vs. 89.6%, *p* = 0.78) between VEPE and CPE TKA. KSS function score resulted significantly higher in the VEPE group over CPE (77 vs. 63, *p* = 0.01). RLLs were more frequent in VEPE than CPE (54% vs. 32%, *p* = 0.05), mainly noticed medially and posteriorly beneath the tibial plate, adjacent to the trochlear shield and the posterior condyles. Osteolysis was observed in one knee per group, but patients were asymptomatic with stable implants.

**Conclusion:**

TKA with VEPE and CPE tibial inserts showed comparable survival rates, complications and clinical and radiographic results up to 10‐year follow‐up.

**Level of Evidence:**

Level III.

**Abbreviations**
AP: anterior‐posterior
CI: confidence interval
CPE: conventional polyethylene
FJS‐12: Forgotten Joint Score
HXLPE: highly cross‐linked ultra‐high molecular weight polyethylene
KSS: American Knee Society Score
RLLs: radiolucent lines
SD: standard deviation
TKA: total knee arthroplasty
VEPE: vitamin E‐stabilized polyethylene

## INTRODUCTION

One of the most common reasons for late failure after total knee arthroplasty (TKA) remains implant aseptic loosening [29]. Highly cross‐linked ultra‐high molecular weight polyethylene (HXLPE) has been introduced attempting to reduce tibial insert wear and particle‐induced osteolysis responsible for implant failure due to wear‐related aseptic loosening in the long term [17]. To date, the superiority of HXLPE over conventional polyethylene (CPE) in terms of revision rate, aseptic loosening and osteolysis has not been proven by clinical studies [4, 12], but a significantly lower revision rate is shown in the Australian Joint Replacement Registry [29].

Concerns regarding the fewer mechanical properties and the oxidative degradation risk of HXLPE tibial inserts have contributed to the use of vitamin E‐stabilize HXLPE also in TKA after its introduction in total hip arthroplasty [2, 5, 13, 25]. The first vitamin E‐stabilized HXLPE polyethylene used for knee inserts was the vitamin E‐infused HXLPE developed by Biomet, E1 (Zimmer‐Biomet), introduced in 2007 for hips and in 2008 for knees [23]. Today, the use of HXLPE, with or without vitamin E, is constantly increasing in TKA as alternative bearing, mostly in young, active and more demanding patients who are at higher risk of revision in the long term [15, 22, 29].

So far, there is still a relative paucity of clinical results in the literature regarding vitamin E‐stabilized HXLPE in TKA [3, 9, 18]. Recently, two clinical studies reported comparable results at short to midterm between TKA performed with or without vitamin E using the same knee design [10, 33]. A systematic review reported similar outcomes of antioxidant‐stabilized HXLPE in TKA as compared to CPE and HXLPE up to midterm [31].

Since the clinical relevance of vitamin E‐stabilized polyethylene (VEPE) with regard to CPE is still unclear in TKA and there is a lack of long‐term results, this study aimed to investigate if there were any differences in survival rate and clinical and radiological results between TKA performed with the same knee design using VEPE or CPE tibial insert at minimum 7‐year follow‐up. The study hypothesis was that there were no differences in survival rates between VEPE and CPE TKA.

## METHODS

The present study was an observational, comparative, cross‐sectional investigation of a retrospective cohort. Inclusion criteria were primary TKA for any indication, minimum 7‐year follow‐up and same knee prosthetic design with VEPE or CPE tibial inserts. No exclusion criteria were set for patient selection. Those patients who underwent TKA in the same orthopaedic centre between January 2011 and December 2015 and who met these criteria were identified as eligible.

The study was approved by the local ethics committee in June 2021, protocol number CE142/21, and the protocol has been registered in ClinicalTrials.gov (NCT05810285). The study was conducted in accordance with the ethical standards laid down by the 1964 Declaration of Helsinki and its later amendments [35]. All patients enrolled gave their written informed consent.

The primary study end point was defined as the cumulative probability of implant survival with revision for aseptic loosening as the end point. The study aimed to test the non‐inferiority of VEPE TKA in terms of implant survival with revision for aseptic loosening in comparison to CPE TKA. Secondary study end points were implant survival with revision for any reason, any other complications, reoperations, revisions, clinical outcomes and radiographic results.

### Implant description

**Figure 1:**
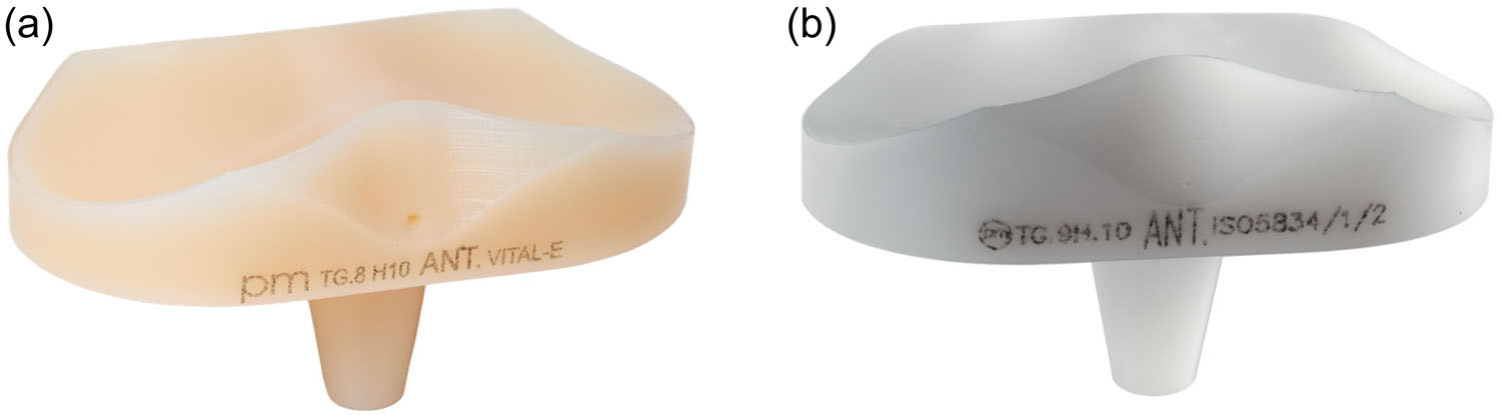
Vitamin E‐blended ethylene oxide sterilized polyethylene (a) and conventional polyethylene (b) ultra‐congruent rotating tibial inserts implanted with the same cemented rotating platform titanium‐niobium nitride coated bicompartmental knee prosthesis in all investigated knees.

The investigated prosthetic implant was a cemented rotating platform bicompartmental knee prosthesis, fully coated with titanium‐niobium nitride (GKS Prime Flex Mobile Bioloy®, Permedica Orthopaedics). All knees included in the study received the same knee prosthesis, with VEPE or CPE ultra‐congruent rotating articular insert (Figure 1). VEPE was GUR1020 blended with 0.1% vitamin E (alpha‐tocopherol) before compression moulding, not cross‐linked and terminally ethylene oxide sterilized (VitalE®). This antioxidant‐stabilized polyethylene was developed in 2009 in collaboration with the Chemistry Department of the University of Turin, Italy. CPE was compression moulded GUR1020, not highly cross‐linked and 25 kGy E‐beam sterilized.

### Surgical procedure

All surgeries were performed by the same team headed by the senior surgeon (M.S.). The same surgical procedure was used in both groups. Spinal anaesthesia was commonly used or general anaesthesia in case of spinal deformities. Tranexamic acid was intravenously administered before surgery and topically applied when needed. A tourniquet was not applied in any procedure. After an anterior longitudinal straight skin incision was performed over the patella, the knee was exposed through medial parapatellar arthrotomy. Mechanical alignment was used for femoral and tibial osteotomies with 5° of valgus in relation to the femoral anatomical axis. All prosthetic components were cemented with non‐antibiotic loaded radiopaque bone cement (LIMA CMT‐1, LimaCorporate, San Daniele del Friuli), usually applied only beneath the tibial plateau and femoral condyles. All arthroplasties were performed without patella resurfacing. The choice to use VEPE or CPE tibial insert was upon the senior surgeon's decision based on the patients' conditions. VEPE was preferably used for younger and active patients.

### Study procedure

A search on the hospital database was carried out to identify eligible patients, using search term references of the investigated implant. All medical records were selected from the hospital database and reviewed for those patients who met all inclusion criteria. Patient contact data, age at operation, gender, BMI, diagnosis, date of intervention, surgical procedure and any following reintervention or revision surgery were registered. All identified TKAs were then divided into two groups according to the type of tibial insert polyethylene VEPE and CPE as the control group.

A preliminary telephone interview was performed with all identified patients. Patients were asked for informed consent and agreement to take part in the study, to collect their current clinical condition and any complication or reoperation postoperatively occurred. Patients were then invited for a clinical and radiographic follow‐up visit at the same centre.

### Clinical assessment

Clinical assessment was carried out at the last follow‐up through the Forgotten Joint Score (FJS‐12) [1]. FJS‐12 was taken at the first telephone call with recruited patients. Patients were asked to answer all 12 issues about their current quality of life and operated knee conditions. Patients were also asked to choose the level of their usual physical activity level. Patients were then evaluated through the standard American Knee Society Score (KSS), knee and function during the follow‐up visit [14].

### Radiographic assessment

Radiographic analysis was performed on the radiographs taken immediately after the operation, at the first‐year follow‐up and at the last study follow‐up visit. The anterior‐posterior (AP) and lateral radiographs of the operated knee were independently evaluated by two observers (A.C. and M.B.) who were blinded regarding the type of tibial insert. Any disagreement regarding radiographic evaluation was then resolved by further evaluation of one senior orthopaedic surgeon (D.M.) to achieve consensus for all considered radiographic parameters. All available radiographs were assessed looking for evidence of periprosthetic radiolucent lines (RLLs) and osteolysis. RLLs were described as less than 2 mm thick non‐progressive lucent areas partially present adjacent to bone cement or prosthesis and limited by a radio‐dense line of thick cancellous bone, according to Smith et al. and Goodfellow et al. [30, 34]. Osteolysis was defined as an area of localized progressive bone resorption or endosteal erosion with more than 2 mm thick progressive RLLs extending in a *ballooning* fashion [30]. The presence of RLL or osteolysis was reported for each group and each periprosthetic zone according to the ‘Modern Knee Society Radiographic Evaluation System and Methodology for Total Knee Arthroplasty’ [20].

### Failures and complications

Any intraoperative and postoperative complication, revision and reintervention were recorded for all TKAs. Revision was defined as one or more prosthetic components' surgical removal and replacement. Revision for aseptic loosening was defined as prosthetic loosening in the absence or the presence of osteolysis due to macroscopic polyethylene wear [6]. Reintervention was defined as surgical knee reoperation without removing any prosthetic component.

### Study population characteristics

Three hundred fifty eligible TKAs (334 patients) were initially identified from database search, of which 181 (52%) knees (171 patients) received VEPE insert and 169 (48%) knees (163 patients) received CPE insert. Non‐simultaneous bilateral TKA was performed in 10 patients in the VEPE group and in 6 patients in the CPE group, while 7 patients received non‐simultaneous TKA with both VEPE and CPE inserts. Overall, 151 (43%) implants (79 VEPE and 72 CPE knees) were lost to follow‐up because 147 patients were unreachable by phone or mail and then were excluded from the study, leaving a cohort of 186 patients, 199 (57%) knees, available for the analysis, with 102 VEPE insert (VEPE group) and 97 CPE insert (CPE group).

**Figure 2:**
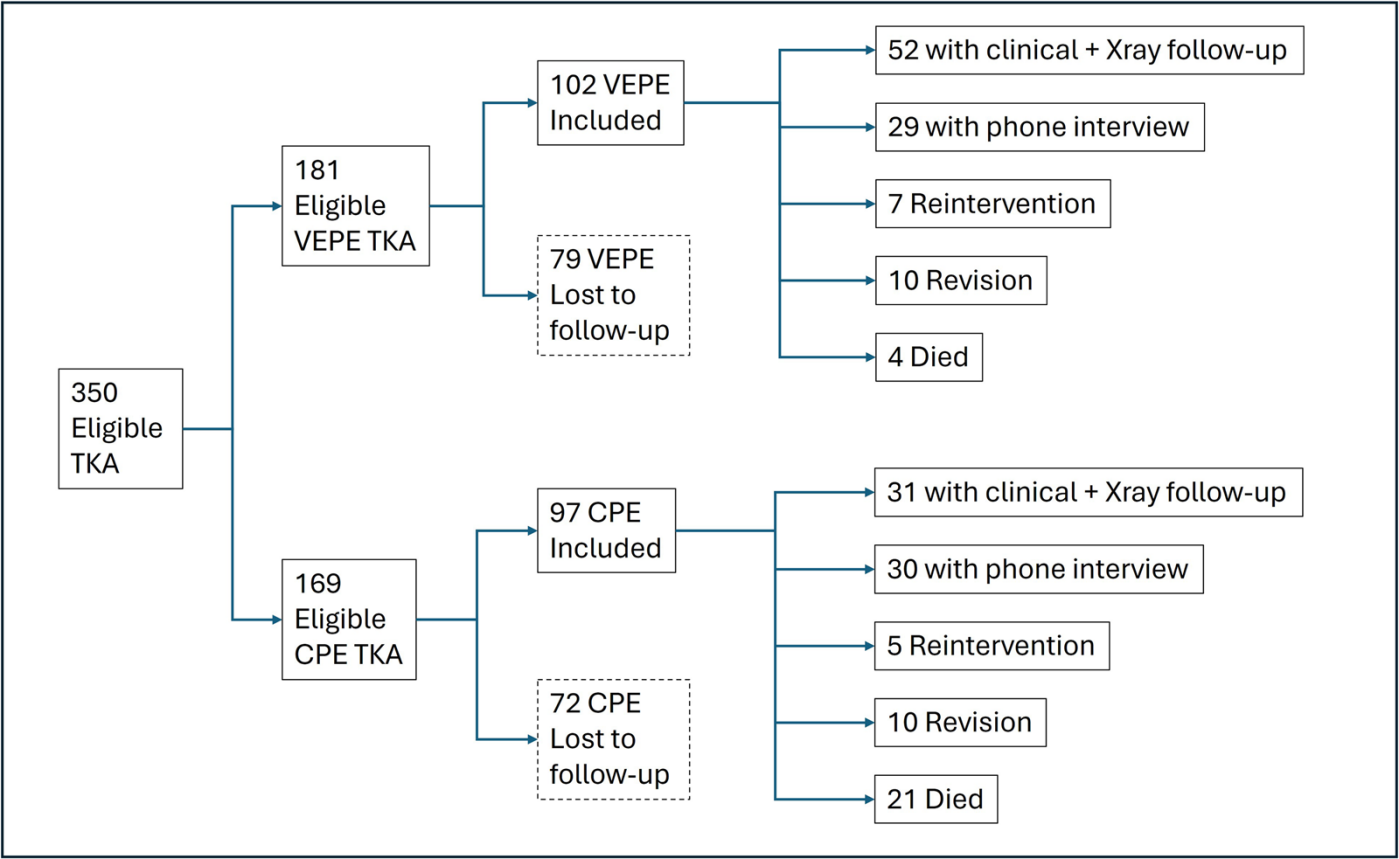
Study flowchart. Out of the 350 eligible TKAs initially identified, 79 VEPE and 72 CPE knees were found lost to follow‐up and were excluded from the study. Analysis regarded the remaining 102 VEPE and 97 CPE knees. CPE, conventional polyethylene; FU, follow‐up; TKA, total knee arthroplasty; VEPE, vitamin E‐stabilized polyethylene.

Within the included cohort of 199 TKAs, 131 patients, 142 (71%) implants (81 VEPE and 61 CPE knees), were found available for follow‐up after first contact by phone call, of whom 83 (42%) implants (52 VEPE and 31 CPE) were assessed with clinical examination, KSS, FJS‐12 and radiographic follow‐up and 59 (29%) implants (29 VEPE and 30 CPE) only with FJS‐12 filled during phone interview. 32 patients, 32 (16%) implants (17 VEPE and 15 CPE knees), were found reoperated. 24 patients, 25 (13%) implants (4 VEPE and 21 CPE knees), deceased of causes unrelated to their implant and none of these patients had been revised before their death, as documented by medical records and by contact with their family, so they were considered as censored events in survival analysis. The study flowchart is summarized in Figure 2.

### Statistical analysis

The study was designed as a non‐inferiority study for binary variables with paired groups to calculate the population sample size. Our hypothesis was that there would be no relevant difference in survival rate for aseptic loosening at mid to long term between the same design VEPE and CPE tibial inserts. Referring to 98.7% and 98.6% as survival rates for loosening at 10‐year follow‐up for CPE TKA [29], with a non‐inferiority margin of 4% deemed as clinically relevant difference in survival rate between paired groups at mid to long‐term follow‐ups [29], sample size calculation gave 99 cases required per group. Cumulative probability of implant survival was analyzed according to Kaplan–Meier method for each group defining revision for aseptic loosening and revision for any reason as the end points. Log‐rank test was performed for significant differences in survival between groups. 95% confidence intervals were reported. Chi‐square and Fisher's exact tests were performed to test significant differences for dichotomous variables. Student's *t* test was used to test significant differences for continuous variables between groups with normal distribution, while the Mann–Whitney *U* test was used when the distribution was not normal. Data distribution was tested for normality with Shapiro–Wilk and D'Agostino Pearson tests. Kappa coefficient was calculated to test interobserver agreement for qualitative radiographic evaluation of periprosthetic RLLs and osteolysis. Statistical significance was set with *p* value ≤ 0.05. All statistical analyses were performed using data analysis and visualization software (GraphPad Prism 10.1.0 Software).

## RESULTS

The mean follow‐up of the VEPE and CPE groups was 8.5 years (range 0.1 [early failure] to 10.9) and 8.3 years (range 0.1–10.3), respectively. Characteristics of the included patients are summarized in Table 1.

**Table 1:** Patient characteristics of the eligible study population. The information about the patient's usual physical activity level has been reported only for 81 knees with VEPE and 61 knees with CPE.

Group	VEPE	CPE	*p* Value
No. of knees (No. of patients)	102 (95)	97 (91)	‐
Mean follow‐up (SD; range)	8.5 (2.1; 0.1–10.9)	8.3 (2.2; 0.1–10.3)	0.3299
Diagnosis, No. of knees	‐	‐	
Osteoarthritis	98	96	0.1928
Rheumatoid arthritis	2	0	0.1657
Post‐traumatic osteoarthritis	2	1	0.5905
Gender (male:female)	41:61	17:80	0.0004*
Mean age at surgery (SD; range)	65.3 (6.7; 42–85)	75.3 (5.7; 60–88)	<0.0001*
Mean BMI (SD; range)	29.9 (5.9; 20–43)	27.8 (5.1; 18–42)	0.0373*
Usual physical activity, No. of knees	81	61	‐
Intense (outdoor activities, sports, heavy works)	7	1	0.0732
Normal (activity of daily living)	45	30	0.4513
Reduced (sedentary life, limited walks)	28	26	0.3277
Almost none (limited movements at home)	1	4	0.0885

Abbreviations: BMI, body mass index; CPE, conventional polyethylene; SD, standard deviation; VEPE, vitamin E‐stabilized polyethylene.

* *p* < 0.05.

**Figure 3:**
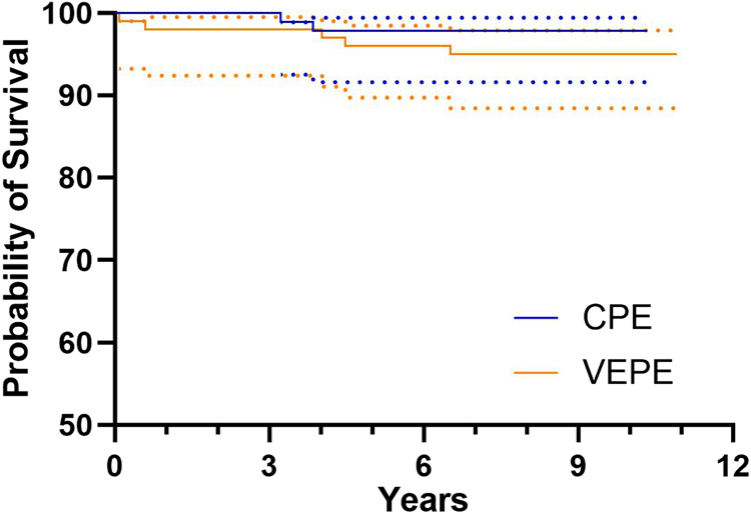
Survival rates with revision for aseptic loosening as the end point for VEPE versus CPE groups. Dotted lines are 95% confidence intervals. CPE, conventional polyethylene; VEPE, vitamin E‐stabilized polyethylene.

**Figure 4:**
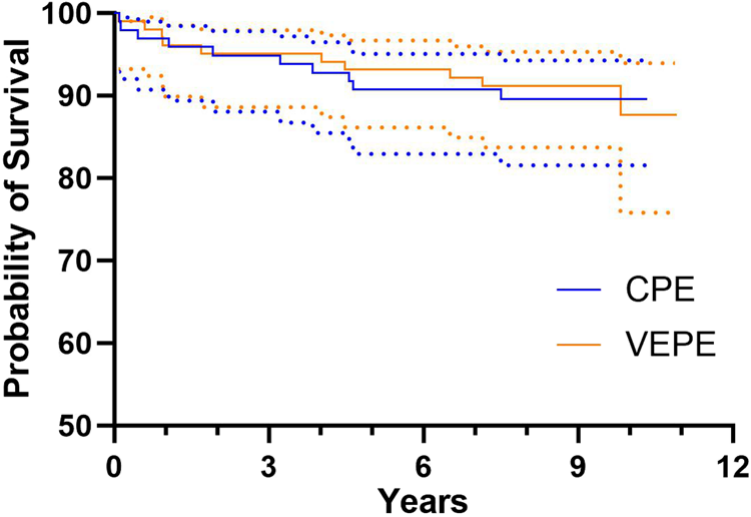
Survival rates with revision for any reason as the end point for VEPE versus CPE groups. Dotted lines are 95% confidence intervals. CPE, conventional polyethylene; VEPE, vitamin E‐stabilized polyethylene.

There were 10 (9.8%) revisions in the VEPE group, of which 5 (4.9%) were due to aseptic loosening, and 10 (10.3%) revisions in the CPE group, of which 2 (2.0%) were due to aseptic loosening (*p* = 0.9057 and *p* = 0.4458, respectively). The mean time to revision for aseptic loosening was 3.1 and 3.5 years after the index procedure for VEPE and CPE, respectively (*p* = 0.8571). No significant differences were found in survival rates with revision due to aseptic loosening (95.0% vs. 97.8%, *p* = 0.2944) or due to any reason (87.6% vs. 89.6%, *p* = 0.7831) between VEPE and CPE at 10‐year follow‐up (Figures 3 and 4, Table 2).

**Table 2:** Study results reported for VEPE versus CPE groups.

Group	VEPE (102 knees)	CPE (97 knees)	*p* Value
No. of revisions for any reason	10 (9.8%)	10 (10.3%)	0.9057
No. of revisions for aseptic loosening	5 (4.9%)	2 (2.0%)	0.4458
Mean time to revision for aseptic loosening (SD; range)	3.1 (2.7; 0.1–6.5)	3.5 (0.4; 3.2–3.8)	0.8571
No. of revisions for other reasons	5 (4.9%)	8 (8.2%)	0.3984
	Of which: 4 for pain 1 other	Of which: 2 infections 2 periprosthetic fractures 2 for pain 2 insert dislocations	
No. of reinterventions for patella resurfacing	4 (3.9%)	2 (2.0%)	0.6833
No. of reinterventions for other reasons	3 (2.9%)	3 (3.1%)	1.0000
	Of which: 2 stiffness 1 debridement	Of which: 1 patella fracture 1 debridement 1 other	
Survival with revision for any reason [95% CI]	87.6% [75.8–93.8]	89.6% [81.6–94.2]	0.7831
Survival with revision for aseptic loosening [95% CI]	95.0% [88.5–97.8]	97.8% [91.6–99.4]	0.2944
Mean KSS—Knee (SD; range)	84 (16; 44–99)	80 (16; 43–99)	0.3174
Mean KSS—Function (SD; range)	77 (26; 0–100)	63 (31; 0–100)	0.0111*
Mean FJS (SD; range)	59 (30; 0–100)	56 (27; 8–100)	0.3785
No. of complications without reintervention	23 (22.5%)	15 (15.4%)	0.2037
	Of which: 15 stiffness 6 pain 1 intraoperative tibial fracture 1 deep vein thrombosis	Of which: 1 intraoperative patella fracture 2 delayed wound healing 6 pain 4 stiffness 1 instability 1 ventricular tachycardia	

Abbreviations: CI, confidence interval; CPE, conventional polyethylene; FJS, Forgotten Joint Score 12; KSS, Knee Society Score; SD, standard deviation; VEPE, vitamin E‐stabilized polyethylene.

* *p* < 0.05.

Reinterventions were performed in 7 (6.8%) VEPE knees and 5 (5.1%) CPE knees (*p* = 0.7682). Reasons for reintervention were patellar resurfacing (6), stiffness (2), debridement for infection (2) and patella fracture (1). All other perioperative complications which did not involve surgical reintervention are summarized in Table 2.

No significant difference was observed in KSS knee score (84 vs. 80, *p* = 0.3174) and FJS‐12 (59 vs. 56, *p* = 0.3785) between VEPE and CPE, respectively, but significant difference was found in KSS function score (77 vs. 63, *p* = 0.0111).

Patients in the VEPE group were significantly younger (65.3 vs. 75.3, *p* < 0.0001), with higher male percentage (40.2% vs. 17.5%, *p* = 0.0004) and higher body mass index (BMI) (29.9 vs. 27.8, *p* = 0.0373) than the CPE group (Table 1).

Since the baseline characteristics of VEPE and CPE groups were significantly different in age, BMI and male percentage, a 1:1 pair matching for age was then performed with a stratified analysis for survivals in VEPE and CPE subgroups (Table 3). Again, survival rates with revision due to aseptic loosening (93.3% vs. 95.2%, *p* = 0.6706) or any reason as the end‐point (83.3% vs. 84.3%, *p* = 0.5848) did not significantly differ.

**Table 3:** Patient characteristics, survival rates and clinical outcomes for VEPE versus CPE age‐matched subgroups.

Age‐matched subgroup	VEPE	CPE	*p* Value
No. of matched knees (No. of patients)	45 (45)	45 (45)	‐
Mean follow‐up (SD; range)	8.5 (2.4; 0.1–10.9)	8.1 (2.4; 0.5–10.2)	0.0607
Gender (male:female)	17:28	7:38	0.0171*
Mean age at surgery (SD; range)	70.5 (3.6; 67.0–84.6)	70.8 (3.3; 59.6–74.3)	0.1256
Mean BMI (SD; range)	27.7 (4.8; 20.8–36.4)	29.8 (4.9; 22.2–41.7)	0.0737
Survival with revision for any reason [95% CI]	83.3% (63.9–92.8)	84.3% (69.8–92.2)	0.5848
Survival with revision for aseptic loosening [95% CI]	93.3% (80.6–97.8)	95.2% (82.3–98.8)	0.6706

Abbreviations: BMI, body mass index; CI, confidence interval; CPE, conventional polyethylene; SD, standard deviation; VEPE, vitamin E‐stabilized polyethylene.

* *p* < 0.05.

**Figure 5:**
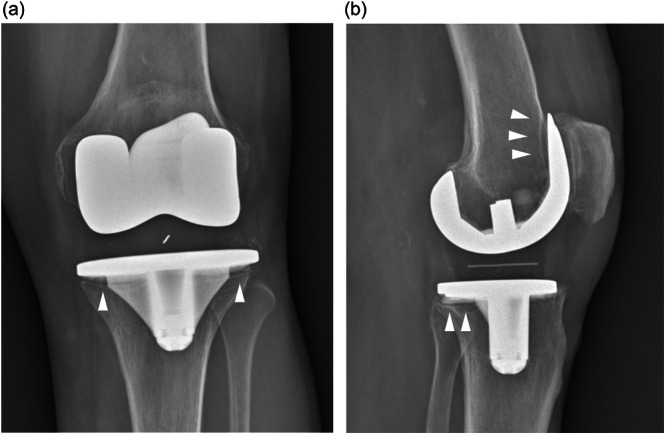
Anterior‐posterior (a) and lateral (b) radiographs of the knee taken at 10‐year follow‐up showing periprosthetic radiolucent lines (white arrowheads) around knee implant with VEPE tibial insert. VEPE, vitamin E‐stabilized polyethylene.

**Figure 6:**
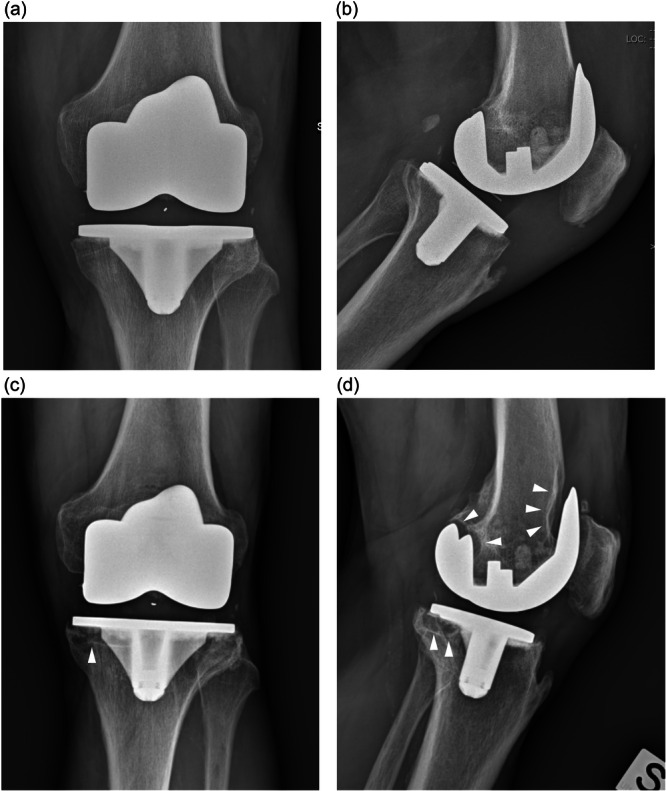
Anterior‐posterior and lateral radiographs of the knee taken immediately postoperatively ((a) and (b), respectively) and at 8‐year follow‐up ((c) and (d), respectively) showing periprosthetic osteolysis (white arrowheads) around knee implant with VEPE tibial insert. VEPE, vitamin E‐stabilized polyethylene.

RLLs were mainly observed with moderate to substantial agreement beneath the medial and posterior sides of the tibial plate and beneath the posterior condyles and trochlear shield of the femoral component in each group. After consensus for all disagreements, RLLs resulted more frequently in the VEPE group (28/52, 54%) in comparison to CPE (10/31, 32%) p = 0.0561, even if patients were all asymptomatic (Figure 5, Table 4). No significant difference was found in clinical scores between patients with and without RLLs, within the same group (Table 5). Periprosthetic osteolysis was found in two knees. One knee with VEPE insert showed extensive bone resorption areas with progressive RLLs >2 mm in zones 1 and 2 around the femoral component and in zone 2 beneath the posterior portion of tibial plate in a 70‐year‐old man with the 8‐year follow‐up (Figure 6). The other knee with CPE insert in a 77‐year‐old woman revealed the same pattern of osteolysis in the same femoral and tibial zones after 10 years. Both patients were completely asymptomatic with stable implants. Neither osteolysis nor macroscopic radiographic signs of polyethylene wear were visible on the pre‐revision radiographs of the aseptic loosening failures.

**Table 4:** Periprosthetic radiolucent lines assessment. The presence of radiolucency is reported by each observer as number and percentage (%) for each periprosthetic zone, as described by Meneghini et al. [20] in VEPE and CPE groups.

		VEPE (52)				CPE (31)				
	Group (knees)	Obs 1	Obs 2	K (Obs Agr)	After Agr consensus	Obs 1	Obs 2	K (Obs Agr)	After Agr consensus	*p* Value
RLL Tibia AP	Zone 1	15 (29%)	15 (29%)	0.719 (0.88)	16 (31%)	6 (19%)	7 (23%)	0.708 (0.90)	5 (16%)	0.1931
	Zone 3M	1 (2%)	0	0 (0.98)	0	0	0	n.a. (1.00)	0	1.0000
	Zone 5	3 (6%)	2 (4%)	0.790 (0.98)	3 (6%)	0	0	n.a. (1.00)	0	0.2895
	Zone 3L	0	0	n.a. (1.00)	0	0	0	n.a. (1.00)	0	1.0000
	Zone 2	7 (13%)	13 (25%)	0.515 (0.84)	8 (15%)	2 (6%)	5 (16%)	0.528 (0.90)	2 (6%)	0.3079
RLL Tibia Lat	Zone 1	6 (12%)	8 (15%)	0.506 (0.88)	5 (10%)	0	1 (3%)	0 (0.97)	0	0.1517
	Zone 3A	4 (8%)	2 (4%)	0.649 (0.96)	3 (6%)	0	0	n.a. (1.00)	0	0.2895
	Zone 5	4 (8%)	4 (8%)	1.00 (1.00)	4 (8%)	0	0	n.a. (1.00)	0	0.2916
	Zone 3P	4 (8%)	3 (6%)	0.276 (0.94)	2 (4%)	0	0	n.a. (1.00)	0	0.5263
	Zone 2	10 (19%)	14 (27%)	0.570 (0.85)	10 (19%)	6 (19%)	8 (26%)	0.817 (0.94)	6 (19%)	1.0000
RLL Femur Lat	Zone 1	5 (10%)	12 (23%)	0.524 (0.87)	9 (17%)	2 (6%)	4 (13%)	0.635 (0.94)	3 (10%)	0.5208
	Zone 3A	1 (2%)	2 (4%)	0.658 (0.98)	1 (2%)	0	0	n.a. (1.00)	0	0.1443
	Zone 5	0	1 (2%)	0 (0.98)	0	0	0	n.a. (1.00)	0	1.0000
	Zone 3P	1 (2%)	3 (6%)	0.485 (0.96)	2 (4%)	1 (3%)	1 (3%)	1.00 (1.00)	1 (3%)	1.0000
	Zone 2	6 (12%)	7 (13%)	0.912 (0.98)	7 (13%)	5 (16%)	3 (10%)	0.716 (0.94)	5 (16%)	0.7555

*Note*: *p* Value was analyzed for results found after agreement consensus between VEPE and CPE.

Abbreviations: Agr, agreement; AP, anterior‐posterior; CPE, conventional polyethylene; K, Kappa coefficient; Lat, lateral; n.a., not applicable; Obs, observer; VEPE, vitamin E‐stabilized polyethylene.

**Table 5:** Clinical scores comparison for patients with and without radiolucency lines (RLLs).

	VEPE (52)			CPE (31)		
Score mean (SD, range)	RLL yes (28, 54%)	RLL no (24, 46%)	*p* Value	RLL yes (10, 32%)	RLL no (21, 68%)	*p* Value
KSS knee	85.36 (17.08, 46–99)	86.42 (14.19, 55–99)	0.5488	88.3 (11.38, 67‐–99)	78.90 (17.18, 43–99)	0.1282
KSS function	83.21 (19.87, 40–100)	76.88 (20.89, 45–100)	0.2091	72.00 (22.75, 35–100)	63.33 (24.10, 35–00)	0.3489
FJS‐12	58.98 (29.61, 2.1–100)	58.00 (28.64, 0–100)	0.9052	64.97 (25.01, 25–100)	47.40 (21.91, 14.6–100)	0.0555

*Note*: Mean values, SD, and range of clinical scores were reported and analyzed for knees with at least one RLL (‘RLL yes’ subgroup) or none (‘RLL no’ subgroup) within the VEPE and CPE groups.

Abbreviations: CPE, conventional polyethylene; FJS‐12, Forgotten Joint Score; KSS, Knee Society Score; SD, standard deviation; VEPE, vitamin E‐stabilized polyethylene.

## DISCUSSION

In the present study, we found no significant difference between VEPE and CPE groups in terms of all‐cause and aseptic loosening survival rates.

Our revisions for aseptic loosening were likely not related to polyethylene wear, but rather were caused by loss of fixation, since the short time to revision, the lack of osteolysis and the lack of radiographic evidence of insert wear. The aetiology of loosening is known to change with time. Loosening reported in the first few years most likely reflects failure to gain fixation, whereas loosening reported in later years is often due to loss of fixation secondary to lysis and bone resorption [29]. Because aseptic loosening and osteolysis are often reported together as failure reasons, we decided to define aseptic loosening for survival endpoint as the combination of loosening and loosening due to osteolysis as described by Brown et al. [6].

Since we found significantly different age, male prevalence and BMI between groups, survival results could have been biased by these covariates, which are known to negatively affect implant survival rate and polyethylene wear [8, 21, 29]. Therefore, we age‐matched the included patients to perform a stratified analysis for survival rates. Again, we found no significant differences in survival rates between VEPE and CPE, even if the subgroup size results were considerably reduced to draw statistically strong conclusions.

No significant difference resulted also in postoperative KSS knee score, FJS‐12, RLLs or osteolysis prevalence. KSS function score resulted significantly higher for VEPE knees, likely due to the younger age and higher male percentage, which might have biased functional outcomes.

A non‐significant higher percentage of asymptomatic RLLs was observed in VEPE knees, mainly around the tibial component in zones 1 and 2 in AP view and zone 2 in lateral view. A possible reason for this finding might again rely on the significantly younger age, higher male percentage and higher BMI of these patients, which could have affected periprosthetic bone remodelling around the tibial component, rather than tibial insert wear. Active and more demanding patients, as younger males, in fact, might be associated with higher stresses beneath the tibial plate, thus explaining the higher number of RLLs in the VEPE group.

To the best of our knowledge, the current literature on TKA with vitamin E‐stabilized HXLPE regards a few retrospective clinical studies reporting outcomes from case series with the same vitamin E‐infused HXLPE up to maximum 7‐year mean follow‐up [9, 10, 33] and retrieval studies investigating material properties and articular surface damages of different brands of antioxidant stabilized HXLPE tibial inserts [19, 26, 27, 32]. Flament et al. reported 100% and 95.7% survival rates for aseptic loosening and all causes, respectively, up to a 6.4‐year follow‐up for 163 cemented TKAs [9]. Ftaita et al. did not report any revision rates but 11 and 10 all‐cause reinterventions, of which 1 revision for loosening per group, for 250 TKAs with vitamin E‐infused HXLPE and 153 with CPE, respectively, after a mean 7‐year follow‐up [10]. Takemura et al. reported no revisions nor osteolysis or RLLs at a 2‐year follow‐up for 100 vitamin E‐infused HXLPE and 100 with CPE [33]. No significant difference in revisions for all‐cause or aseptic loosening, nor in clinical or radiographic results, was shown from these comparative clinical studies between vitamin E‐infused HXLPE and CPE [10, 33]. The same conclusions were drawn from the Australian Joint Replacement Registry [29] and from a recent analysis of the American Joint Replacement Registry, in which no difference in all‐cause or aseptic loosening revision rate was found when comparing HXLPE, with or without an antioxidant, to CPE in TKA [16].

The results found in the present study confirmed the current state of the art, with no significant difference in revision rates between antioxidant‐stabilized and non‐stabilized TKA. However, we found a large number of aseptic loosening revisions caused by loss of fixation, in the absence of wear‐related osteolysis and more RLLs in VEPE TKA, probably due to the patient baseline characteristics, in comparison to similar clinical studies [9, 10, 33].

The investigated VEPE in this study has theoretically no residual free radicals inside since no high‐energy irradiation occurs during its manufacturing process. Gas‐sterilized polyethylene inserts indeed have shown no detectable levels of free radicals or oxidation after a variable shelf‐life period [7]. Therefore, there should be no reason to justify antioxidant stabilization with vitamin E in this non‐cross‐linked, ethylene oxide‐sterilized polyethylene. However, besides high‐energy radiations, other mechanisms can also occur and contribute to initiating and accelerating the oxidation process, such as in vivo mechanical stress forces and body fluids absorption due to cyclic loading acting on the polyethylene articular components [24, 28].

Because of the theoretical lack of residual free radicals inside the non‐cross‐linked, ethylene oxide‐sterilized VEPE considered in this study, a lower revision rate due to late wear‐related aseptic loosening could be expected in the long term in comparison to CPE. Wearing debris from the same kind of VEPE should not elicit osteolytic biological response, as previously demonstrated in vitro [11].

Nonetheless, neither the present study nor the available clinical studies have been able up now to demonstrate the superiority of vitamin E‐stabilized polyethylene tibial inserts in terms of revision rates, periprosthetic RLLs or osteolysis in comparison to non‐stabilized inserts, despite the beneficial effects of vitamin E against oxidation, as shown by several in vitro and retrieval studies. Likely, longer follow‐up studies with more accurate and precise methods, such as radiostereometric analysis, are needed to determine any possible benefit of VEPE in reducing tibial liner wear in TKA.

### Study limitations

This study had some limitations. First, since the study was designed as observational, no specific criteria were assigned to use VEPE or CPE insert, but upon the senior surgeon's decision, no patient selection according to patient characteristics was performed for the control group. Thus, the retrospective cohort included non‐homogeneous groups in terms of age, gender and BMI. These covariates have likely biased the results, leading to significantly better knee function and nonsignificant higher RLL prevalence in the VEPE group. Despite the younger age, higher BMI and higher male prevalence in the VEPE group, which means worse conditions for implant survivorship, the survival rates of the VEPE group were not significantly lower in comparison to CPE. To adjust the significantly different baseline characteristics of the cohorts, a pair matching for age was then performed to obtain comparable groups. However, even if the stratified analysis confirmed no difference in aseptic and all‐cause survival rates between VEPE and CPE subgroups, the matched subgroups size was considerably reduced causing limited evidence in the results. Second, the number of patients lost to follow‐up was very high, likely due to the COVID‐19 pandemic situation when the study was ongoing. For this reason, although the initial eligible cohort size was deemed large enough according to the statistical sample size calculation, the number of patients available for complete analysis was significantly reduced. Third, being a cross‐sectional study, preoperative KSS was lacking, leading to the impossibility of comparing knee and function score improvements between groups. Fourth, regarding the radiographic evaluation, some lateral views of knee radiographs showed components not perfectly positioned parallel to the X‐ray beam, so RLLs could not be reliably detected. Therefore, RLLs prevalence could probably be underestimated due to some missing RLLs.

## CONCLUSIONS

This clinical study showed similar survival rates, complications, clinical results and RLLs between VEPE and CPE tibial inserts up to mid to long‐term follow‐up. Stabilizing CPE with blended vitamin E does not directly affect implant survival rate for aseptic loosening or all causes, nor RLLs or osteolysis occurrence in TKA up to 10‐year follow‐up. In conclusion, studies with longer follow‐ups are needed to observe potential vitamin E benefits for reducing all‐cause or aseptic loosening revision rates in TKA.

## AUTHOR CONTRIBUTIONS

**Alessandro Bistolfi**: Conceptualization; methodology; formal analysis; writing—original draft; writing—review and editing; supervision. **Marco Spezia**: Conceptualization; methodology; investigation; writing—review and editing. **Alessandra Cipolla**: Investigation; data curation; formal analysis; writing—review and editing. **Monica Bonera**: Investigation; data curation; formal analysis; writing—review and editing. **Danilo Mellano**: Investigation; data curation; writing—review and editing. **Lorenzo Banci**: Conceptualization; methodology; funding acquisition; project administration; writing—original draft; writing—review and editing; visualization; supervision. **Marta Colombo**: Data curation; formal analysis; project administration; writing—review and editing. **Alessandro Massè**: Conceptualization; writing—review and editing; supervision.

## CONFLICT OF INTEREST STATEMENT

Lorenzo Banci and Marta Colombo are employed as clinical researchers at Permedica Orthopaedics. Marco Spezia is a paid consultant and received research support from Permedica Orthopaedics. The other authors have no relevant financial or non‐financial interests related to the present study to disclose.

## ETHICS STATEMENT

This study was performed in line with the principles of the Declaration of Helsinki. Approval was granted by the Ethics Committee 'Comitato Etico Interaziendale di Novara' on 11 June 2021, with protocol number CE142/21. All patients enrolled in the study gave their written informed consent.

## Data Availability

All data requests should be submitted to the corresponding author for consideration.
